# Norwegian trauma care: a national cross-sectional survey of all hospitals involved in the management of major trauma patients

**DOI:** 10.1186/s13049-014-0064-0

**Published:** 2014-11-12

**Authors:** Oddvar Uleberg, Ole-Petter Vinjevoll, Thomas Kristiansen, Pål Klepstad

**Affiliations:** Department of Emergency Medicine and Pre-Hospital Services, St. Olav’s University Hospital, Trondheim, Norway; Department of Circulation and Medical Imaging, Norwegian University of Science and Technology (NTNU), Trondheim, Norway; Department of Surgery, St. Olav’s University Hospital, Trondheim, Norway; Department of Anesthesiology, Vestre Viken HF, Buskerud Hospital, Drammen, Norway; Department of Anesthesiology and Intensive Care Medicine, St. Olav’s University Hospital, Trondheim, Norway

**Keywords:** Epidemiology, Injury, Norway, Trauma, Trauma system

## Abstract

**Background:**

Approximately 10% of the Norwegian population is injured every year, with injuries ranging from minor injuries treated by general practitioners to major and complex injuries requiring specialist in-hospital care. There is a lack of knowledge concerning the caseload of potentially severely injured patients in Norwegian hospitals. Aim of the study was to describe the current status of the Norwegian trauma system by identifying the number and the distribution of contributing hospitals and the caseload of potentially severely injured trauma patients within these hospitals.

**Methods:**

A cross-sectional survey with a structured questionnaire was sent in the summer of 2012 to all Norwegian hospitals that receive trauma patients. These were defined by number of trauma team activations in the included hospitals. A literature review was performed to assess over time the development of hospitals receiving trauma patients.

**Results:**

Forty-one hospitals responded and were included in the study. In 2011, four trauma centres and 37 acute care hospitals received a total of 6,570 trauma patients. Trauma centres received 2,175 (33%) patients and other hospitals received 4,395 (67%) patients. There were significant regional differences between health care regions in the distribution of trauma patients between trauma centres and acute care hospitals. More than half (52.5%) of the hospitals received fewer than 100 patients annually. The national rate of hospital admission via trauma teams was 13 per 10,000 inhabitants. There was a 37% (from 65 to 41) reduction in the number of hospitals receiving trauma patients between 1988 and 2011.

**Conclusions:**

In 2011, hospital acute trauma care in Norway was delivered by four trauma centres and 37 acute care hospitals. Many hospitals still receive a small number of potentially severely injured patients and only a few hospitals have an electronic trauma registry. Future development of the Norwegian trauma system needs to address the challenge posed by a scattered population and long geographical distances. The implementation of a trauma system, carefully balanced between centres with adequate caseloads against time from injury to hospital care, is needed and has been shown to have a beneficial effect in countries with comparable challenges.

## Background

The Global Burden of Injury Study reported a 9.3% reduction in deaths caused by injuries from 1990 until 2010; however, traumatic injury is still recognized as one of the primary challenges in modern health care [1,2]. Every year, approximately 5.1 million deaths worldwide are caused by injuries of any type, which represent a mortality rate of 74 per 100,000 persons and constitute the leading cause of death from 1 to 44 years of age [1,3]. The Norwegian mortality rate related to trauma varies among reports, with rates ranging from 29 to 77 per 100,000, depending on which definitions are used [4-10]. In Norway, approximately 540,000 persons are injured annually [8], 36,000 persons sustain permanent functional impairment, 1,200 persons receive disability pensions [8,11], and approximately 2,500 persons die as a result of accidents and violence, including self-inflicted injuries [8,11].

Several publications have shown a beneficial effect with the implementation of trauma systems in terms of reduced morbidity and mortality [12-16]. Trauma systems advocate both preventative measures aimed at reducing the incidence of traumatic injuries, and pre- and in-hospital clinical efforts to reduce mortality and morbidity [12]. Several trauma models have been described, and the optimal organization of trauma care hospitals may be different in countries with a scattered population, such as Norway, compared with more populated areas [16,17].

In 2007, a national report on the current status of trauma services proposed the implementation of a national trauma system for Norway [18]. Hospitals receiving trauma patients should be organized into two levels and the regional health trusts decided as a policy that each health region should have one coordinating trauma centre. One university hospital in each region should act as the trauma centre and have the formal responsibility for regional trauma organization [7,18]. The other acute care hospitals should either provide initial stabilisation before transfer or definite trauma care [7,18]. Trauma centres should provide definite care for all injuries. Still, some hospitals, not defined as trauma centres, are equally able to provide trauma centre level of care [18-21].

The 2007 national trauma report showed that 71% (34/48) of hospitals received fewer than 100 trauma patients per year and that the majority of Norwegian hospitals treated few seriously injured patients [18]. Norwegian health care is in constant change. Therefore, the report published in 2007 may not represent the current number of hospitals involved in trauma care and the number of received patients per hospital. Thus, the aim of the study was to describe the current status of the Norwegian trauma system by identifying the number and the distribution of contributing hospitals and the caseload of potentially severely injured trauma patients within these hospitals.

## Methods

### Study setting

Norway has a scattered population and a low population density (15 inhabitants per km^2^) [22]. The Norwegian mainland covers 324,000 km^2^, with a straight-line distance of 1,800 km from north to south [22]. In 2011, Norway had a total population of 4,920,305 [23]. Previously, the responsibility of regional specialist health services, including hospital care, was provided by 19 counties. In 2002, this responsibility was assumed by five newly formed regional health authorities (RHA), which were reduced to four RHAs in 2007 [24]. As described in the national trauma report in 2007, 48 acute care hospitals nationwide received potentially severely injured patients, and the population covered by each hospital ranged from 13,000 to 2,500,000 [18].

All hospitals have predefined trauma teams, though the activation criteria show considerable variation among hospitals [22]. Criteria describing trauma transfers from acute care hospitals to trauma centres are generally lacking [25]. The pre-hospital emergency service is well established and consists of dispatch centres/emergency medical communication centres (EMCC), ground ambulances, on-call primary care doctors and air ambulances [22]. The helicopter service in the national air ambulance service consists of 12 primary air ambulance helicopters, which are manned with a pilot, an anaesthesiologist and a paramedic/rescuer [26]. Six search and rescue helicopters operated by the Royal Norwegian Air Force perform regularly ambulance missions and are also staffed with an anaesthesiologist as an integrated part of the national air ambulance services [26]. The health system is publicly funded and the Norwegian health legislation emphasises the importance of equal access for all citizens to adequate health care, regardless of residential pattern [24].

### Study design

The study was conducted as a cross-sectional survey. The hospitals were identified through an overview of Norwegian hospitals provided by the National Directorate of Health and were included in the study if they A) had an emergency department, and B) had 24-hour acute surgical services [27]. In July 2012, a structured questionnaire was sent by electronic mail to each hospital’s trauma coordinator. The questionnaire contained questions regarding the availability of a local electronic trauma registry and the number of trauma patients treated by trauma teams at their facility in 2011. A trauma patient/potential severely injured patient was defined as a patient receiving trauma team attendance, according to the hospital’s trauma team activation (TTA) protocol [22]. Where applicable, number of patients who were transferred among hospitals was also included if this resulted in a TTA [22]. The hospitals that had no system for registration of potentially severely injured patients were asked to estimate the number of patients, based on other sources of information (e.g., manual counting of trauma charts and/or number of performed CT trauma protocols). If the hospital did not respond or if the answers were inconclusive, a follow-up telephone interview was conducted with the hospital trauma coordinator.

Information concerning time trends in hospital trauma care was obtained from an unstructured search of Norwegian scientific articles and white paper reports describing the Norwegian hospital acute care services.

### Ethics

The Regional Committee for Medical and Health Research Ethics was informed about the study and decided that formal ethical approval was not required (REC Central Norway 2014/763).

### Statistical analysis

Descriptive data are presented as absolute numbers, percentages and ranges, where appropriate. We used Pearson’s chi-squared test to compare observations from different health regions. *P* < 0.05 was considered to be statistically significant. Data analysis was performed using statistical software (IBM Corp., released 2012. SPSS Statistics for Windows, Version 21.0.0.2, IBM Corporation, Armonk, NY, USA).

## Results

Forty-one hospitals responded and were included in the study. A total of 6,570 trauma patients were admitted to four trauma centres and 37 acute care hospitals. Of these, 4,722 (72%) were exact figures based on the data in the trauma registries and 1,848 (28%) were estimated from other sources. Thirteen hospitals reported the existence of a local electronic trauma registry.

**Figure 1:**
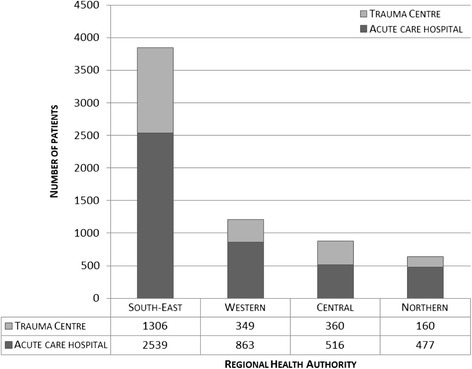
**Distribution of patients per region and type of hospital.**

One third of the patients (*n* = 2,175; 33%) were admitted to a trauma centre, and two-thirds (*n* = 4,395; 67%) were admitted to acute care hospitals (Figure 1). The relative contribution from trauma centres in different regions ranged from 25% (Northern RHA) to 41% (Central RHA). Corresponding figures in Western RHA was 29% and 34% in South-East RHA, respectively. Comparing regions among each other, there were significant differences between three of four regions (p <0.05), except between Western and Northern RHA (p =0.10).

More than half of the hospitals (52.5%) received fewer than 100 trauma patients (Table 1).

**Table 1:** **Distribution of patients within hospitals categorized by number of received patients**

**Categorization of hospitals by number of received patients**	**Number of hospitals (%)**	**Total number of received patients (%)**
≤ 100	21 (52.5)	1,182 (18)
101 - 300	14 (35)	2,586 (39)
> 300	5 (12.5)	2,802 (43)
**TOTAL**	40* (100)	**6,570 (100)**

*Two hospitals reported a collective number of patients.

**Figure 2:**
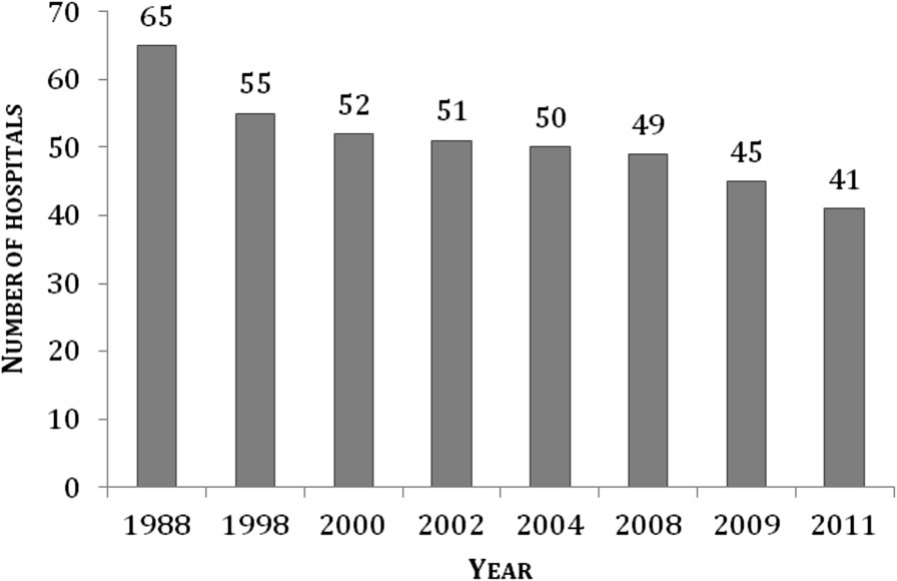
**Number of Norwegian hospitals receiving trauma patients.**

**Figure 3:**
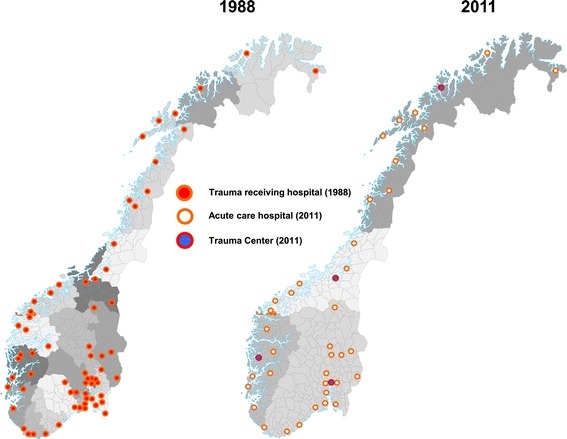
**Overview of Norwegian hospitals receiving trauma patients in 1988 and 2011.**

The national rate of trauma admission was 13 per 10,000 inhabitants (Table 2). The total number of patients varied between health care regions (Figure 1); when adjusted for population, the admission rates per 10,000 inhabitants were similar in all regions (range 12–14) (Table 2). We found five articles and two white paper reports, in addition to our own findings (*n* = 41), regarding the number of hospitals receiving trauma patients [9,22,28-32]. There was a 37% (from 65 to 41) reduction in the number of hospitals involved in acute trauma care between 1988 and 2011 (Figures 2 and 3).

**Table 2:** **Regional characteristics and trauma patients in different health regions per 10,000 inhabitants**

	**South-East RHA**	**Western RHA**	**Central RHA**	**Northern RHA**	**Norway**
Population	2,743,875	1,028,069	680,110	468,251	4,920,305
Area (km^2^)	111,012	43,439	56,385	112,946	323,782
Inhabitants per km^2^	25	24	12	4	15
Number of patients	3,845	1,212	876	637	6,570
Number of hospitals	17	7	7	10	41
Patients per 10,000 inhabitants	14	12	13	14	13

RHA: Regional Health Authority.

## Discussion

Within the last two decades, there has been a substantial reduction in the number of Norwegian hospitals receiving potentially injured patients. Many hospitals still receive a small number of trauma patients, and only few hospitals have an electronic trauma registry. The number of trauma patients differs substantially among the four health regions, but the rates are similar when adjusted for population size. The acute care hospitals receive two thirds of all trauma patients and make a substantial contribution within the Norwegian trauma system. The distribution of patients between trauma centres and acute care hospitals shows regional variation.

In our study, we found an estimated 6,570 patients who were suspected of having a potential severe injury after accidents and who required specialist health care. Compared with the total national number of injured patients (*n* = 540,000), only a minor number of patients are considered potentially severely injured in the initial phase after injury [8]. The definition of severe injury/major trauma is internationally recognized as having an injury severity score (ISS) above 15 (ISS >15) [33,34]. In our study we chose to include patients receiving trauma team activation, in order to try to describe the overall workload of potential severely injured patients in Norwegian hospitals. TTA is performed when potential severe injury is anticipated [35]. To register ISS would have given more information about the patients’ severities of injuries and the potential over-triage; however, this was not possible as many hospitals lack data on ISS [10,18]. Previous reports from Norwegian university hospitals have reported the rate of trauma patients having TTA with an ISS lower than 15 to be from 71% to 78%, corresponding to a high number of over-triage [21,36,37]. Applying these rates to our findings (*n* = 6,570), the total number of severely injured patients (ISS >15) is in the range of approximately 1,400 to 1,900 per year. These numbers are comparable to trauma care in Scotland (approximately 1,100 severe trauma cases per year/population of 5.2 million) and Finland (approximately 1,000-1,300 severe trauma cases per year/population of 5.3 million) [38,39]. A Norwegian study by Hansen et al. found the incidence of severe injury (ISS >15) in the western part of Norway to be 30 per 100,000 corresponding to 1,476 severely injured patients in Norway every year [4]. Notably, the study by Hansen et al. also included pre-hospital deaths [4].

Several studies and white paper reports have in the period from 1988 to 2011 described the number of hospitals receiving trauma patients (Figure 2) [9,22,28-32]. These and our findings show a 37% reduction in Norwegian hospitals receiving trauma patients (Figures 2 and 3) [9,22,28-32]. In 2005, Wisborg and colleagues found that in Scandinavia, the number of receiving hospitals ranged from 41 to 60 hospitals (except Iceland with two hospitals) within countries that had comparable populations and health system structures [40]. The high number of hospitals within each country leads to challenges with a low caseload of severe injuries for many hospitals. Fewer cases reduce the experience for each hospital’s trauma teams and potentially result in poorer clinical outcomes [40]. In our study, we observed that although many hospitals still receive relatively few patients, the rate of hospitals receiving less than 100 patients is reduced from 71% (2007) to 53% (2011) [18]. The actual threshold in the volume of trauma patients needed to maintain sufficient quality in trauma care is debated [41]. North American recommendations for the needed volume of trauma patients range from 200 to 650 severely injured patients (ISS >15) or each surgeon should treat more than 35 patients with ISS >15 [42-44]. Although a certain minimum in volume of trauma patients is needed to achieve sufficient experience, geography, residential pattern and structure of special national health services are also important factors for each country’s or region’s organization of trauma care [38].

In our study we also found that there is an uneven admission rate between the regions in the number of patients transported directly to the trauma centres versus acute care hospitals (Figure 1). These differences may be caused by different organizational structure, geography and number of contributing hospitals. In the northern RHA a low initial admission rate (25%) to the regional trauma centre can be due to long distances, low population density and challenging weather conditions. Therefore, the initial admissions may often be at the closest local hospital. The relatively higher admission rate in the central RHA (41%) may be due to a smaller geographically defined area making transport directly to the trauma centre more feasible (Table 2). In the western RHA, potentially severely injured patients are admitted to two university hospitals with all surgical specialities, whereas only one is formally defined as a trauma centre. This resulted in a low trauma centre admission rate (29%) [19,20].

In a study by Kristiansen et al. that included 8,466 trauma deaths in a 10-year period, they observed a significantly higher mortality rate in rural areas compared to more urban areas. Additionally, they found that 78% of trauma deaths occurred outside the hospital [9]. This might imply that designing a trauma system for a country such as Norway with large rural areas, based on trauma models developed in highly urbanised areas, may be suboptimal. The establishment of a regionalised inclusive trauma system in Victoria, Australia showed significantly better functional outcomes and reduced mortality [16,45]. Adopting elements from well-documented trauma systems in regions with similar population and geographical characteristics may be advantageous for the Norwegian trauma system [10,16,45-47]. An inclusive trauma model contains all elements of trauma care from the pre-hospital phase, through hospital treatment both in trauma and non-trauma centres, and to end of rehabilitation. The exclusive systems focus primarily on trauma centres and their capabilities [17]. In a study by Utter et al. a 23% mortality reduction in an inclusive trauma system was observed compared to the more exclusive systems [17].

Challenges facing Norwegian trauma care are relatively many hospitals with a low caseload of severely injured patients, harsh climatic conditions and long geographic distances. A tendency towards centralisation has been observed, although there may be a lack of fully developed inclusive regional trauma systems [7]. Targeted resources must be allocated if Norway intends to maintain a geographically dispersed network of competent trauma hospitals. Implementing an inclusive trauma model in this setting would mean a continued effort in integrating all elements of care from adequate pre-hospital response, in-hospital treatment to rehabilitation. Potentially severely injured should early be triaged to the closest available hospital, capable of managing their injuries [10,19,21]. This should be based on common triage guidelines and well-educated pre-hospital personnel [10,19,21]. The hospitals should be accredited according to available trauma resources and should provide services according to predefined roles in trauma care. The national air emergency services has a compensating effect to adjust for geographical dispersion and potential unequal access to advanced emergency medical care. However, the service is subjected to seasonal (e.g. weather conditions) and operational challenges which may reduce the all-year reliance of the service. This is something the trauma system needs to be aware of when allocating trauma resources [28,48].

An implicit need in a well-designed mature trauma system is the availability of data on the incidence and distribution of injury, operational characteristics of the trauma system and functional outcome as provided by a quality registry [15]. In our study, we found that only 32% (13/41) of included hospitals had an electronic trauma registry. Previous investigations have found that there is no uniform reporting among these registries [10]. While some hospitals have used the trauma registry provided by the BEST initiative, some of the university hospitals have developed their own solutions [10,49]. The widespread lack of trauma care registrations in Norway is an obstacle against developing the optimal national trauma care system [8].

We recognise that this survey has several limitations. First, the present study collected data primarily by obtaining information from one contact person at each hospital. The responses were not validated, e.g., by interviewing other persons within the same hospital. Another limitation is that the number of trauma patients may be overestimated because some patients are initially received at local hospitals and are later transferred to trauma centres for definitive care [22]. Finally, the estimated the number of trauma patients in different hospitals may be influenced by different definitions used to identify trauma patients [22].

## Conclusion

In 2011, acute hospital trauma care in Norway was delivered by four trauma centres and 37 acute care hospitals. This number of participating hospitals has been reduced by 37% since 1988. However, many hospitals still receive a small number of patients and only a few hospitals have an electronic trauma registry. Future development of the Norwegian trauma system needs to address the challenge posed by a scattered population and long geographical distances that influence timely access to definitive care. The implementation of a trauma system, carefully balanced between centres with adequate caseloads against time from injury to hospital care, is needed and has been shown to have a beneficial effect in countries with comparable challenges.

## References

[1] Lozano R, Naghavi M, Foreman K, Lim S, Shibuya K, Aboyans V, Abraham J, Adair T, Aggarwal R, Ahn SY, Alvarado M, Anderson HR, Anderson LM, Andrews KG, Atkinson C, Baddour LM, Barker-Collo S, Bartels DH, Bell ML, Benjamin EJ, Bennett D, Bhalla K, Bikbov B, Bin Abdulhak A, Birbeck G, Blyth F, Bolliger I, Boufous S, Bucello C, Burch M (2012). Global and regional mortality from 235 causes of death for 20 age groups in 1990 and 2010: a systematic analysis for the Global Burden of Disease Study 2010. Lancet.

[2] Søreide K (2009). Epidemiology of major trauma. Br J Surg.

[3] World Health Organization: **World Report on Road Traffic Injury Prevention.** In Geneva: World Health Organization; 2004.

[4] Hansen KS, Morild I, Engesæter LB, Viste A (2004). Epidemiology of severly and fatally injured patients in western part of Norway. Scand J Surg.

[5] Lund J (2004). Epidemiology, Registration and Prevention of Accidental Injuries. PhD thesis.

[6] Lund J, Bjerkedal T (2001). Permanent impairments, disabilities and disability pensions related to accidents in Norway. Accid Anal Prev.

[7] Kristiansen T (2013). Epidemiology and Management of Traumatic Injuries: A Population-Based Study of Fatal Trauma an Assessment of Geographical Challenges in the Organization of Trauma Care. PhD thesis.

[8] Norwegian Institute of Public Health: **The Panorama of Injuries in Norway – Emphasis on Injury in Central Registries.** In [http://www.fhi.no/dokumenter/8558040d0a.pdf] (accessed 14^th^ July 2014).

[9] Kristiansen T, Lossius HM, Rehn M, Kristensen P, Gravseth HM, Roislien J, Soreide K (2014). Epidemiology of trauma: a population-based study of geographical risk factors for injury deaths in the working-age population of Norway. Injury.

[10] Kristiansen T, Søreide K, Ringdal KG, Rehn M, Krüger AJ, Reite A, Meling T, Naess PA, Lossius HM (2010). Trauma systems and early management of severe injuries in Scandinavia: review of the current state. Injury.

[11] Norwegian Government. Ministry of Health and care services: **Accidents in Norway - the National Strategy for the Prevention of Accidents Involving Personal injury (2009–2014). Report No I-1146. In Norwegian.** In [http://www.regjeringen.no/upload/HOD/Dokumenter%20FHA/I-1146%20Ulykker%20i%20Norge.pdf] (accessed 14^th^ July 2014).

[12] American College of Surgeons Committee on Trauma: **Resources for Optimal Care of the Injured Patient.** In Chicago, IL: American College of Surgeons; 2006.

[13] MacKenzie EJ, Rivara FP, Jurkovich GJ, Nathens AB, Frey KP, Egleston BL, Salkever DS, Scharfstein DO (2006). A national evaluation of the effect of trauma-center care on mortality. N Engl J Med.

[14] Twinjstra MJ, Moons KG, Simmermacher RK, Leenen LP (2010). Regional trauma system reduces mortality and changes admission rates: a before and after study. Ann Surg.

[15] Ruchholtz S, Lefering R, Paffrath T, Oestern HJ, Neugebauer E, Nast-Kolb D, Pape HC, Bouillon B (2008). Reduction in mortality of severely injured patients in Germany. Dtsch Arzteblatt Int.

[16] Cameron PA, Gabbe BJ, Cooper DJ, Walker T, Judson R, McNeil J (2008). A statewide system of trauma care in Victoria: effect on patient survival. Med J Austr.

[17] Utter GH, Maier RV, Rivara FP, Mock CN, Jurkovich GJ, Nathens AB (2006). Inclusive trauma systems: do they improve triage or outcomes of the severely injured?. J Trauma.

[18] **National working group, Report on organization on treatment of seriously injured patients - Trauma system.** In Oslo, South-East Regional Health Trust: Norwegian; 2007. [https://ekstranett.helse-midt.no/1001/Sakspapirer/sak%2025-12%20vedlegg%201%20Traumesystem%20i%20Norge%20-%20Forslag%20tl%20organisering%20av%20behandling%20av%20alvorlig%20skadde%20pasienter.pdf] (accessed 14^th^ July 2014).

[19] Søreide K, Krüger AJ, Vårdal AL, Ellingsen CL, Søreide E, Lossius HM (2007). Epidemiology and contemporary patterns of trauma deaths: changing place, similar pace, older face. World J Surg.

[20] Søreide K (2010). Temporal patterns of death after trauma: evaluation of circadian, diurnal, periodical and seasonal trends in 260 fatal injuries. Scand J Surg.

[21] Rehn M, Lossius HM, Tjosevik KE, Vetrhus M, Østebø O, Eken T, Rogaland Trauma System Study Collaborating Group (2012). Efficacy of a two-tiered trauma team activation protocol in a Norwegian trauma centre. Br J Surg.

[22] Larsen KT, Uleberg O, Skogvoll E (2010). Differences in trauma team activation criteria among Norwegian hospitals. Scand J Trauma Resusc Emerg Med.

[23] *Statistics Norway.* [https://www.ssb.no/statistikkbanken/selectvarval/Define.asp?SubjectCode=01&ProductId=01&MainTable=NY3026&SubTable=Kommun1&PLanguage=0&Qid=0&nvl=True&mt=1&pm=&gruppe1=KommNyeste&gruppe2=Hele&gruppe3=Hele&aggreg1=NO&aggreg2=&aggreg3=&VS1=Kommun&VS2=Kjonn&VS3=AlleAldre00B&CMSSubjectArea=befolkning&KortNavnWeb=folkemengde&StatVariant=&TabStrip=Select&checked=true] (accessed 14^th^ July 2014).

[24] Norwegian Government. Ministry of health and care services: **National Health and Care Plan 2012–2015.** Norwegian: [http://www.regjeringen.no/en/dep/hod/documents/regpubl/stmeld/2010-2011/meld-st-16-20102011.html?showdetailedtableofcontents=true&id=639794] (accessed 14^th^ July 2014).

[25] Kristiansen T, Lossius HM, Søreide K, Steen PA, Gaarder C, Næss PA (2011). Patients referred to a Norwegian Trauma Centre: effect of transfer distance on injury patterns, use of resources and outcomes. J Trauma Manag Outcomes.

[26] The Norwegian Air Ambulance Foundation: **Capacity and Base Structure – A report on the Norwegian Air Ambulance Service 1988 – 2011.** In September 2013. [http://cdn.norskluftambulan.netdna-cdn.com/wp-content/uploads/2013/09/SNLA-Kapasitet-ogbasestruktur-rapport-sept2013.pdf] (accessed: 8^th^ of July 2014).

[27] The Norwegian Directorate of Health: **Overview of National Health Institutions.** In [http://www.frittsykehusvalg.no] (accessed 12^th^ March 2012).

[28] Ringen AH, Hjortdahl M, Wisborg T (2011). Norwegian trauma team leaders–training and experience: a national point prevalence study. Scand J Trauma Resusc Emerg Med.

[29] Brattebo G, Wisborg T, Høylo T (2001). Organization of trauma admissions at Norwegian hospitals. Tidsskr Nor Laegeforen.

[30] Isaksen MI, Wisbrog T, Brattebo G (2006). Organisation of trauma services–major improvements over four years. Tidsskr Nor Laegeforen.

[31] SINTEF/NIS (Norwegian institute for hospital research) (1990). The Air Ambulance Services in Norway; A Report Based on Activity Data from Fiscal year 1988.

[32] Norwegian Government. Ministry of health and care services: **If it is Urgent….. Requirements for Emergency Medical Preparedness. NOU 1998:9**. Norwegian: [http://www.regjeringen.no/Rpub/NOU/19981998/009/PDFA/NOU199819980009000DDDPDFA.pdf] (accessed 14^th^ July 2014).

[33] Lossius HM, Rehn M, Tjosevik KE, Eken T (2012). Calculating trauma triage precision: effects of different definitions of major trauma. J Trauma Manag Outcomes.

[34] Baker SP, O’Neill B, Haddon W, Long WB (1974). The injury severity score: a method for describing patients with multiple injuries and evaluating emergency care. J Trauma.

[35] Georgiou A, Lockey DJ (2010). The performance and assessment of hospital trauma teams. Scand J Trauma Resusc Emerg Med.

[36] Uleberg O, Vinjevoll OP, Eriksson U, Aadahl P, Skogvoll E (2007). Overtriage in trauma – what are the causes?. Acta Anaesthesiol Scand.

[37] Dehli T, Fredriksen K, Osbakk SA, Bartnes K (2011). Evaluation of a university hospital trauma team activation protocol. Scand J Trauma Resusc Emerg Med.

[38] Jansen JO, Campell MK, on behalf of the GEOS investigators (2014). The GEOS study: designing a geospatially optimised trauma system for Scotland. Surgeon.

[39] Handolin L, Leppäniemi A, Vihtonen K, Lakovaara M, Lindahl J (2006). Finnish trauma audit 2004: current state of trauma management in finnish hospitals. Injury.

[40] Wisborg T, Castren M, Lippert A, Valsson F, Wallin CJ, Working Scandinavian Group (WISE) (2005). Training trauma teams in the Nordic countries: an overview and present status. Acta Anaesthesiol Scand.

[41] Søreide K (2014). Effect of regional trauma centralization on volume, injury severity and outcomes of injured patients admitted to trauma centres. Br J Surg.

[42] Smith RF, Frateschi L, Sloan EP, Campbell L, Krieg R, Edwards LC, Barrett JA (1990). The impact of volume on outcome in seriously injured trauma patients: two years' experience of the Chicago Trauma System. J Trauma.

[43] Nathens AB, Jurkovich GJ, Maier RV, Grossman DC, MacKenzie EJ, Moore M, Rivara FP (2001). Relationship between trauma center volume and outcomes. JAMA.

[44] American College of Surgeons: **Rescources for Optimal Care of the Injured Patient: 1999. 1999 ed.** In Chicago: American College of Surgeons; 1998.

[45] Gabbe BJ, Simpson PM, Sutherland AM, Wolfe R, Fitzgerald MC, Judson R, Cameron PA (2012). Improved functional outcomes for major trauma patients in a regionalized, inclusive trauma system. Ann Surg.

[46] Atkin C, Freedman I, Rosenfeld JV, Fitzgerald M, Kossmann T (2005). The evolution of an integrated State Trauma System in Victoria, Australia. Injury.

[47] Liberman M, Mulder DS, Lavoie A, Sampalis JS (2004). Implementation of a trauma care system: evolution through evaluation. J Trauma.

[48] Haug B, Avall A, Monsen SA (2009). Reliability of air ambulances - a survey in three municipalities in Helgeland?. Tidsskr Nor Laegeforen.

[49] Hestnes M (2004). The trauma registry Ullevål University Hospital. Scand J Trauma Resusc Emerg Med.

